# AI-Based Structured Information Extraction From Synthetic Nursing Handover Transcripts: Comparative Evaluation of Large Language Models

**DOI:** 10.2196/106133

**Published:** 2026-10-05

**Authors:** Aaron Conway, Adriana Hada, Jessica Schluter, Hui (Grace) Xu, Dan Lowden, Tim Miller, Ken Donald, Andrew Teodorczuk

**Affiliations:** 1 Centre for Healthcare Transformation, Queensland University of Technology, Park Rd, Kelvin Grove, Brisbane, QLD, 4059, Australia, 61 731383887; 2 School of Nursing, Queensland University of Technology, Brisbane, Australia; 3 The Prince Charles Hospital, Metro North Health, Brisbane, Australia; 4 Caboolture Hospital, Brisbane, Australia; 5 School of Electrical Engineering and Computer Science, The University of Queensland, Brisbane, Australia; 6 School of Medicine & Dentistry, Griffith University, Gold Coast, Queensland, Australia; 7 The University of Queensland Northside Clinical Unit, The University of Queensland, Brisbane, Australia; 8 Royal Australian and New Zealand College of Psychiatrists, Melbourne, Australia

**Keywords:** artificial intelligence, clinical handover, nursing, patient handoff, natural language processing, large language models, prompt engineering, information extraction, patient safety, machine learning

## Abstract

**Background:**

Clinical handover is the process during which responsibility and accountability for care are transferred between clinicians. AI has the potential to improve the reliability and completeness of clinical handover by helping clinicians detect predefined content areas that have been communicated, identify explicit information gaps, and prompt clarification before responsibility is transferred.

**Objective:**

This study evaluated the performance of several large language models and prompt optimization strategies for structured information extraction of synthetic nursing handover transcripts.

**Methods:**

Two registered nurses independently annotated a dataset of 203 synthetic handover transcripts to produce consensus labels for information extraction tasks. Tasks included (1) labeling spans of text into SBAR (Situation, Background, Assessment, Recommendation) categories, (2) content detection to determine if specific pieces of information were communicated, and (3) labeling spans of text that communicated information using uncertain terms that included a subtask for identifying unknown facts. Baseline and Genetic-Pareto (GEPA)–optimized prompts were compared for the GPT-5.2, GPT-5-nano, and MedGemma 27B large language models. Additionally, the LangExtract framework was evaluated for span-extraction tasks.

**Results:**

The GPT-5.2–optimized model achieved a micro*–F*_1_-score of 0.85 (95% CI 0.83‐0.88) for content detection, an absolute improvement of +0.08 compared with the matched baseline. GPT-5-nano also performed better after optimization for content detection (micro*–F*_1_-score 0.81, 95% CI 0.78‐0.84), suggesting that this structured task was not limited to the highest-capacity model. For SBAR span extraction, GPT-5.2 with prompt optimization achieved a micro*–F*_1_-score of 0.76 (95% CI 0.72‐0.79), improving by +0.24 compared with baseline and exceeding LangExtract; GPT-5-nano also improved to a micro*–F*_1_-score of 0.69 (95% CI 0.66‐0.72). Broad uncertainty-span extraction remained comparatively weak despite prompt optimization (micro*–F*_1_-score 0.41, 95% CI 0.33‐0.48; absolute improvement +0.06). In contrast, explicit unknown-fact extraction was more accurate with GPT-5.2 (micro*–F*_1_-score 0.84, 95% CI 0.63‐1.00), GPT-5-nano (micro*–F*_1_-score, 0.84 95% CI 0.63‐1.00), and MedGemma 27B (micro*–F*_1_-score 0.80, 95% CI 0.63‐1.00). Genetic-Pareto–optimized prompts outperformed the LangExtract approach across each span-extraction task.

**Conclusions:**

Prompt optimization improved matched-model point estimates, with the highest performance observed for predefined content detection and SBAR span extraction. Broad uncertainty extraction remained less accurate than the narrower unknown-fact task. These technical results do not establish clinical effectiveness, safety, or readiness for real-time use. Validation using authentic nursing handover communication and prospective evaluation in clinical workflows are required before clinical application.

## Introduction

Clinical handover is the process during which responsibility and accountability for some or all aspects of a patient’s care are transferred to another clinician or clinical team [1]. In nursing, shift-to-shift and transfer handovers support continuity of surveillance, care priorities, pending actions, and the inclusion of patient and family concerns. Inaccurate, incomplete, or misinterpreted communication at this transition can contribute to delays, duplicated work, and preventable harm [2,3]. International and Australian patient-safety standards therefore prioritize structured clinical handover, particularly at shift changes, transfers, and discharge [1,4].

Structured handover processes aim to standardize minimum content and the format of exchange so that critical information is predictably conveyed, acted upon, and auditable. In Australia, the Communicating for Safety Standard emphasizes structured clinical handover while retaining opportunities for questions, clarification, and confirmation [1,5]. One widely used framework is SBAR (Situation, Background, Assessment, Recommendation), which organizes clinician-to-clinician handover content into 4 information categories [6]. Recent systematic review evidence suggests that structured handoff protocols may improve some safety outcomes, but the certainty and implementation fidelity vary by protocol and setting; evidence specific to SBAR remains low certainty [7].

Even where structured tools are mandated or encouraged, their enactment in nursing handovers remains shaped by ward-level organizational and cultural conditions, time demands, interruptions, and the need to communicate patient-specific information for continuity of care [8,9]. This variability is amplified in bedside nursing handover where patient/family involvement is increasingly emphasized, yet research highlights tensions between standardization (predictability) and tailoring (patient-centeredness), along with barriers related to confidentiality and clinician concerns [10,11]. Reviews of handover mnemonics similarly emphasize local validation, clarification, and readback rather than assuming that every element is universally relevant [6].

Traditional supervised information-extraction methods can be highly effective for stable tasks with sufficiently large, task-specific labeled datasets. However, there are several potential advantages of using large language models (LLMs) in this context. For example, the same instruction-driven model can be configured for heterogeneous, context-dependent tasks. This may be useful where annotated nursing handover data are limited and communication is conversational and nonlinear. Prior work has demonstrated that few-shot clinical information extraction with LLMs can be accurate, while also showing that performance depends materially on prompt design and task framing [12,13].

Recent advances in LLMs create an opportunity to support clinical handover by analyzing information as it is communicated. Other studies have examined AI-based approaches to support clinical handover, although evaluations of these technologies remain limited [14]. For example, a recent multihospital study used an LLM to pregenerate content to support the preparation for handover [15]. An alternative application of AI yet to be investigated is to support the verbal clinician-to-clinician exchange itself. This spoken exchange remains central to transferring responsibility of care in clinical settings and provides opportunities to question, clarify, and confirm information [1,9]. Accurate extraction of structured information from the spoken exchange is a prerequisite for developing this form of communication support. The aim of this study was to evaluate the accuracy of LLM structured information extraction from synthetic clinical handover transcripts.

## Methods

### Study Design

This study used a model-evaluation design, corresponding to the “LLM evaluation” research-design category in TRIPOD-LLM (Transparent Reporting of a Multivariable Prediction Model for Individual Prognosis or Diagnosis–Large Language Models) [16] (Checklist 1). This category covers studies that assess existing LLMs for their accuracy or suitability for a specific health care task. We compared selected LLMs and prompt configurations on predefined multilabel classification and span-extraction tasks using completed synthetic nursing handover transcripts and registered nurse annotations. The study was not an evaluation in a health care setting because it did not test workflow integration or clinical, administrative, or workforce outcomes.

Tasks related to clinical handover communication that were considered potentially augmentable with AI assistance were identified by the researchers using a co-design process with clinicians and consumers, which will be reported separately. The tasks were as follows:

- Extracting spans of text from transcripts that aligned with the SBAR framework for structuring clinical handover. In this study, SBAR span extraction was operationalized as a sequence-labeling task to identify contiguous spans of text in handover transcripts that corresponded to each SBAR category. This task evaluated whether models could map conversational handover text to SBAR-labeled spans, rather than whether the transcripts themselves followed a clean sequential SBAR structure.
- Identifying if key elements were communicated in handover transcripts as a content detection task. In this study, this task was operationalized as a checklist of items that are recommended to be addressed during nursing clinical handover, which were developed as part of quality-improvement processes at the researchers’ institution. The checklist items are summarized in Table 1.
- Identifying spans of text in transcripts that were communicated using uncertain terms. In this study, this task was operationalized as span annotation of utterances during handover that conveyed incomplete knowledge, vague or hedged wording, imprecise timing, second-hand sourcing, unclear procedures, or unclear responsibility for follow-up actions. These forms of uncertainty were treated as potentially clinically important because they may indicate information that requires clarification or verification by the receiving clinician. The uncertainty categories and example guidance provided to annotators are summarized in Table 2.

**Table 1.** Checklist items used to operationalize identification of key concepts and entities in handover transcripts.

Category	Checklist item
Patient involvement	Clinician introductions Introduction of clinicians to the patient or carer Invitation for the patient or carer to participate in handover
Identification	Verification of 3 patient identifiers
Situation	Primary diagnosis or reason for admission Significant events or complications Current status, including pending tests/procedures and interim plans/orders
Background	Relevant clinical and social history, including comorbidities Falls risk Pressure injury risk Allergies Advance care planning
Assessment	Observations, deterioration score, and recent escalations Pain management Devices, lines, and vascular access Critical monitoring and alarms Nutrition and dietary restrictions Fluid balance and fluid restrictions Infusions Medication chart review, including high-risk medicines Pathology results or pending investigations Mobility and use of aids Skin integrity and related interventions
Recommendation	Discharge plan Critical actions required Follow-up care plan or pathway actions Patient or carer goals and preferences

**Table 2.** Uncertainty categories used to support annotator identification of uncertainty-related spans in handover transcripts.

Category	Definition/when to use	Example from handover speech
Hedge/probability language	The speaker indicates partial confidence or doubt about information.	“I think ENT reviewed him”; “He should be going to theatre soon.”
Vague/qualitative expression	Information is described using imprecise or subjective language.	“He looks fine now”; “Seems okay.”
Unknown fact/explicit lack of knowledge	The speaker openly states missing knowledge or incomplete data.	“Not sure if consent’s been signed”; “I don’t know his allergies.”
Indefinite timing	Timing or schedule for an event is vague or lacks precision.	“Later today”; “After the round.”
Source uncertainty	Information relies on a second-hand or unverifiable source.	“ENT said he’s on the list”; “Night nurse told me.”
Procedural uncertainty	The next step in care is unclear or the plan is not explicitly stated.	“You might want to check his IV.”
Responsibility uncertainty	A required task or follow-up is mentioned, but it is unclear who is responsible for performing it.	“Bloods to be checked later”; “Needs review this afternoon.”

### Data Sources

We used the publicly available National Information and Communications Technology Australia (NICTA) Synthetic Nursing Handover Dataset, which contains synthetic recordings of clinical handovers delivered by a registered nurse based on patient profiles with cardiovascular, neurological, renal, and respiratory conditions [17]. In this dataset, handover monologs were generated from comprehensive patient profiles that included information such as the patient’s name, age, admission history, inpatient duration, and the familiarity between the nurses giving and receiving the handover. The nurse was instructed to simulate a bedside shift-to-shift handover within a medical ward setting [17].

For our study, we used 100 handover samples from the training partition of the NICTA dataset. First, audio recordings from the NICTA dataset were transcribed using the OpenAI Whisper speech-to-text model. Second, 3 videos depicting conversational nursing shift-to-shift handovers were transcribed in a similar manner to provide examples of interactive handover dialogue. These videos were developed at the authors’ institution for educational purposes to demonstrate best practices for clinical handovers that are used in the undergraduate nursing program. Transcripts from the educational videos were then used as few-shot examples within the DSPy framework [18] using the BootstrapFewShot optimizer to guide the transformation of the 100 NICTA monolog transcripts into 2-sided conversational handovers so that the dataset would better reflect real-world contemporary clinical handover interactions. To broaden the range of clinical contexts represented in the dataset, we further synthesized 103 additional handover examples using the GPT-5 model interactively in a chat interface. These scenarios included inter- and intrahospital transfers, postprocedural handovers, emergency department transitions, and handovers that involved patients with complex mental health care needs. The final dataset comprised 203 synthetic handover transcripts used for subsequent annotation and model development.

### Ethical Considerations

Ethics approval was not sought because this was not human research as defined by the National Statement on Ethical Conduct in Human Research [19]. The study involved only synthetic handover transcripts generated for model evaluation, with no recruitment, observation, or testing of human participants, and no use of patient, clinician, clinical-record, personal, identifiable, or potentially reidentifiable data. Accordingly, the study did not require human ethics review, and a formal exemption from ethics review was not applicable.

### Annotation

Two annotators, who are experienced clinically active registered nurses, independently annotated transcripts using a structured rubric. Labeled annotations that met consensus between reviewers were used as the reference labels for downstream model development and evaluation.

Annotation was performed using the Prodigy annotation software using a custom interface that presented each transcript in 3 components [20]. First, annotators highlighted relevant spans of text and assigned labels corresponding to the SBAR framework together with markers of communicative uncertainty, including vagueness, hedging, unknown facts, indefinite timing, source uncertainty, procedural uncertainty, and uncertainty regarding responsibility. Overlapping span labels were permitted where a passage served more than 1 communicative function. Second, annotators completed a multiple-response checklist indicating whether predefined handover elements were present in the transcript.

Both annotators reviewed all transcripts independently within the same annotation environment and were supported by written guidance and examples to promote consistent interpretation of the coding framework. For the creation of the reference standard, a consensus dataset was derived by retaining only those span annotations and checklist items for which both annotators agreed. In practical terms, this meant that only text segments assigned the same label by both reviewers, and only checklist items selected by both reviewers, were carried forward for downstream model development and evaluation.

### LLM Prompt Optimization Methods

Annotated handover transcripts were first partitioned deterministically into optimization (75%) and evaluation (25%) subsets using a fixed random split. Prompt optimization was performed within DSPy [18], which is a Python framework that can be used for prompt optimization using feedback from model outputs to improve task performance. For each task, we defined a task-specific DSPy signature that specified the transcript as input and a constrained structured output. Checklist prediction was formulated as multilabel classification, where the task was to return a list of the items from the checklist that were covered in the transcript. The SBAR and uncertainty-related tasks were formulated as span extraction requiring the model to return verbatim text segments from the transcript together with the appropriate label. The uncertainty-related tasks were further subdivided into a broad uncertainty-span extraction task (which included all uncertainty categories) and a more specific unknown-fact extraction task (which included only spans labeled as unknown facts).

Across the baseline DSPy evaluations, Genetic-Pareto (GEPA)–based DSPy optimization experiments, and LangExtract experiments, we purposively selected 3 underlying language models with different deployment profiles: OpenAI GPT-5.2 as the higher-capacity proprietary model, OpenAI GPT-5-nano as a smaller lower-cost proprietary model [21], and MedGemma 27B as a 27-billion-parameter open-weight model developed for medical tasks [22]. This panel was intended to examine matched prompt effects across contrasting model profiles, not to provide an exhaustive leaderboard of all contemporary LLMs. For DSPy baseline and GEPA runs, the same task model was used before and after optimization so that differences reflected the prompt configuration rather than a change in the underlying model. GPT-5.2 and GPT-5-nano were accessed through cloud-hosted OpenAI API end points; MedGemma 27B was run locally on institutional high-performance computing infrastructure.

GEPA was used to optimize the task prompts [23]. It iteratively evaluated candidate task instructions, combined numeric task scores with natural-language error feedback, and used GPT-5.2 as a separate reflection model to propose revisions. Optimization used 576 scoring calls. Checklist optimization targeted multilabel agreement, while span-task optimization rewarded same-label text overlap using intersection over union (IoU) so that closer boundaries received higher scores. A transcript with neither a reference span nor a predicted span for a target label was treated as a correct negative. The final compiled prompts were fixed before evaluation on the held-out partition. For reporting, span detection performance and boundary overlap were presented separately.

In addition to DSPy prompt optimizations, we conducted separate evaluations using the LangExtract framework, as a prompt-based few-shot structured information extraction approach for the SBAR, uncertainty-span, and unknown-fact span tasks [24]. These experiments used task-specific prompt descriptions together with annotated in-context examples derived from the reference data. We used 10 annotated examples from the training partition as few-shot exemplars, and inference was then performed on the full held-out test partition for each task.

### Data Analysis

Interrater agreement was assessed with Cohen κ and mean IoU among matched spans. Agreement CIs were calculated with 2000 bootstrap resamples of transcript pairs using a fixed random seed.

Performance was measured for the checklist content detection task at the level of individual labels with counts of true positives, false positives, false negatives, and true negatives, together with precision, recall, and *F*_1_-score. Aggregate performance was summarized using micro-averaged (pooled), macro-averaged (unweighted mean), and support-weighted precision, recall, and *F*_1_-score across labels.

For span-extraction tasks, precision, recall, and *F*_1_-score were calculated from predicted spans as binary detection measures, so that these statistics reflected the model’s ability to identify the correct labeled spans. Span-boundary agreement was reported separately using the mean IoU across matched pairs. We additionally calculated per-label descriptive metrics including the number of reference spans, the number of predicted spans, matched-span precision, recall, *F*_1_-score, and mean IoU.

Sampling uncertainty was summarized with 95% CIs calculated using nonparametric bootstrap resampling. For each result, we resampled transcripts with replacement 2000 times using a fixed random seed and recalculated the relevant metric from the pooled counts in each resample. Confidence limits are reported as the 2.5th and 97.5th percentiles of the bootstrap distribution.

## Results

### Preconsensus Interrater Agreement

Cohen κ was 0.75 (95% CI 0.73‐0.76) for checklist decisions, 0.70 (95% CI 0.68‐0.72) for pooled SBAR token-by-label decisions, 0.12 (95% CI 0.08‐0.15) for broad uncertainty, and 0.40 (95% CI 0.18‐0.64) for unknown facts. Among overlapping same-label spans, the mean IoU was 0.86 (95% CI 0.85‐0.87) for SBAR, 0.71 (95% CI 0.61‐0.81) for broad uncertainty, and 0.78 (95% CI 0.56‐0.98) for unknown facts. The relatively high matched-span IoU indicates similar boundaries when both nurses identified the same span type, with disagreement in uncertainty annotations arising mainly over whether and how to label an expression.

### Key Findings

Within-model comparisons showed consistent point-estimate gains with DSPy/GEPA over matched unoptimized-prompt baselines. For GPT-5.2, micro*–F*_1_-score increased from 0.77 (95% CI 0.74‐0.80) to 0.85 (95% CI 0.83‐0.88) for checklist prediction, from 0.51 (95% CI 0.47‐0.55) to 0.76 (95% CI 0.72‐0.79) for SBAR span extraction, from 0.35 (95% CI 0.28‐0.41) to 0.41 (95% CI 0.33‐0.48) for uncertainty span extraction, and from 0.76 (95% CI 0.50‐1.00) to 0.84 (95% CI 0.63‐1.00) for unknown-fact extraction. Across span tasks, LangExtract generally had lower point estimates than the corresponding highest DSPy/GEPA configuration. Among matched span predictions, the mean IoU exceeded 0.8 for the highest-performing GPT-5.2 span-extraction configurations. Figure 1 summarizes matched within-model comparisons across tasks for the 3 evaluated models.

**Figure 1.**
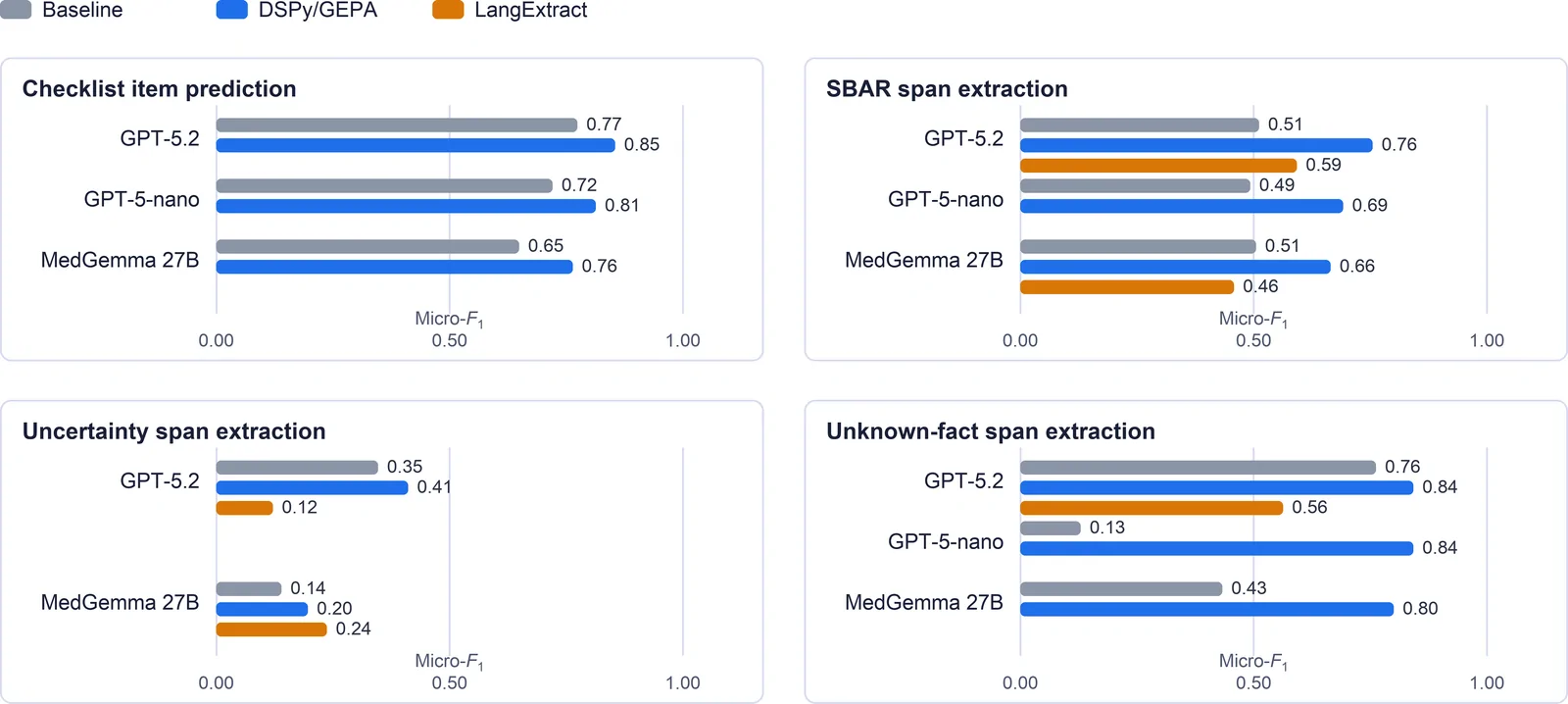
Micro*–F*_1_-scores for baseline, Genetic-Pareto (GEPA)–optimized, and LangExtract strategies for each model within each task.

### SBAR Span Extraction

Among SBAR configurations, the highest overall score was achieved by DSPy/GEPA-optimized GPT-5.2 (micro*–F*_1_-score 0.76, 95% CI 0.72‐0.79), followed by DSPy/GEPA-optimized GPT-5-nano (micro*–F*_1_-score 0.69, 95% CI 0.66‐0.72) and LangExtract GPT-5.2 (micro*–F*_1_-score 0.59, 95% CI 0.56‐0.62). Table 3 provides label-level results for the best-performing GPT-5.2 DSPy/GEPA SBAR configuration.

**Table 3.** Per-label SBAR (Situation, Background, Assessment, Recommendation) metrics for the best-performing GPT-5.2 DSPy/Genetic-Pareto (GEPA) model.

Label	Reference spans	Predicted spans^a^	Recall (95% CI)	Precision (95% CI)	Mean IoU^b^ (95% CI)	*F_1_*-score (95% CI)
ASSESSMENT	194	192	0.77 (0.72‐0.82)	0.78 (0.72‐0.83)	0.77 (0.72‐0.81)	0.77 (0.73‐0.81)
BACKGROUND	47	50	0.72 (0.61‐0.84)	0.68 (0.57‐0.79)	0.66 (0.56‐0.75)	0.70 (0.60‐0.80)
RECOMMENDATION	113	124	0.76 (0.69‐0.84)	0.69 (0.62‐0.78)	0.78 (0.73‐0.83)	0.73 (0.67‐0.78)
SITUATION	73	52	0.68 (0.57‐0.81)	0.96 (0.88‐1.00)	0.82 (0.75‐0.88)	0.80 (0.72‐0.89)

a Predicted spans: model-generated spans mapped back to the transcript.

b IoU: intersection over union for matched span boundaries.

Within this GPT-5.2 SBAR comparison, macro-precision increased from 0.41 (95% CI 0.37‐0.44) to 0.78 (95% CI 0.73‐0.82), macro-recall from 0.69 (95% CI 0.63‐0.75) to 0.73 (95% CI 0.69‐0.78), and macro*–F*_1_-score from 0.49 (95% CI 0.46‐0.53) to 0.75 (95% CI 0.71‐0.79). Span-boundary agreement among matched predictions was strongest for SITUATION and RECOMMENDATION, as shown by the label-level mean IoU estimates in Table 3.

### Checklist Task

For checklist prediction, the best overall result was achieved by DSPy/GEPA-optimized GPT-5.2 (micro*–F*_1_-score 0.85, 95% CI 0.83‐0.88; macro*–F*_1_-score 0.73, 95% CI 0.63‐0.76; and support-weighted *F*_1_-score 0.85, 95% CI 0.82‐0.88). DSPy/GEPA-optimized GPT-5-nano also performed competitively (micro*–F*_1_-score 0.81, 95% CI 0.78‐0.84), while MedGemma 27B reached a micro*–F*_1_-score of 0.76 (95% CI 0.74‐0.79). Tables 4 and 5 present grouped per-label estimates for accuracy, precision, recall, and *F*_1_-score for the best-performing GPT-5.2 checklist model.

**Table 4.** Grouped per-label checklist performance for the best-performing GPT-5.2 DSPy/Genetic-Pareto (GEPA) model.

Checklist item	Accuracy (95% CI)	Precision (95% CI)	Recall (95% CI)	*F*_1_-score (95% CI)
Identification				
ID check of 3 patient identifiers	1.00	1.00	1.00	1.00
Situation				
Primary diagnosis | reason for admission	0.96 (0.90‐1.00)	0.96 (0.89‐1.00)	1.00	0.98 (0.94‐1.00)
Current status (awaiting tests/procedures, on interim orders/plan)	0.65 (0.51‐0.78)	0.73 (0.57‐0.88)	0.71 (0.55‐0.87)	0.72 (0.58‐0.84)
Significant events or complications	0.84 (0.73‐0.94)	0.62 (0.25‐1.00)	0.50 (0.17‐0.83)	0.56 (0.20‐0.80)
Background				
Alerts-allergies	0.92 (0.84‐0.98)	0.89 (0.73‐1.00)	0.89 (0.71‐1.00)	0.89 (0.76‐0.98)
Relevant clinical and social history | comorbidities	0.98 (0.94‐1.00)	0.94 (0.81‐1.00)	1.00	0.97 (0.90‐1.00)
Alerts-falls risk	0.98 (0.94‐1.00)	1.00	0.67	0.80
Alerts-pressure injury risk	0.98 (0.94‐1.00)	1.00	0.67	0.80
Advanced care planning	1.00	1.00	1.00	1.00
Assessment				
Observations | Q-ADDS | recent escalations	0.92 (0.84‐0.98)	0.95 (0.87‐1.00)	0.95 (0.87‐1.00)	0.95 (0.89‐0.99)
Medication chart | flag high risk meds	0.90 (0.82‐0.98)	0.86 (0.71‐0.97)	0.96 (0.87‐1.00)	0.91 (0.81‐0.98)
Devices | lines | vascular access	0.94 (0.88‐1.00)	0.88 (0.75‐1.00)	1.00	0.94 (0.86‐1.00)
Mobility | aids	0.90 (0.80‐0.98)	0.80 (0.61‐0.95)	0.94 (0.80‐1.00)	0.86 (0.72‐0.97)
Pain management	0.94 (0.86‐1.00)	0.89 (0.71‐1.00)	0.94 (0.81‐1.00)	0.91 (0.80‐1.00)
Infusions	0.84 (0.73‐0.94)	0.89 (0.67‐1.00)	0.53 (0.27‐0.79)	0.67 (0.40‐0.86)
Pathology	0.88 (0.78‐0.96)	0.74 (0.53‐0.93)	0.93 (0.79‐1.00)	0.82 (0.67‐0.94)
Nutrition | restrictions	0.88 (0.78‐0.96)	0.70 (0.50‐0.89)	1.00	0.82 (0.67‐0.94)
Fluid balance | restrictions	0.90 (0.80‐0.98)	0.70 (0.38‐1.00)	0.78 (0.44‐1.00)	0.74 (0.44‐0.94)
Skin integrity | interventions	0.90 (0.80‐0.98)	0.55 (0.22‐0.83)	1.00	0.71 (0.36‐0.91)
Critical monitoring | alarms	0.96 (0.90‐1.00)	0.00 (0.00‐0.00)	0.00 (0.00‐0.00)	0.00 (0.00‐0.00)

**Table 5.** Grouped per-label checklist performance for the best-performing GPT-5.2 DSPy/Genetic-Pareto (GEPA) model (continued)^a^.

Checklist item	Accuracy^b^ (95% CI)	Precision (95% CI)	Recall (95% CI)	*F*_1_-score (95% CI)
Recommendation				
Care plan/pathway actions to follow up	0.90 (0.80‐0.98)	0.90 (0.80‐0.98)	1.00	0.95 (0.89‐0.99)
Asked patient/carer about goals and preferences	0.80 (0.67‐0.90)	0.00 (0.00‐0.00)	0.00 (0.00‐0.00)	0.00 (0.00‐0.00)
Discharge plan	0.98 (0.94‐1.00)	0.80 (0.33‐1.00)	1.00	0.89 (0.50‐1.00)
Critical actions required	0.92 (0.84‐0.98)	0.20 (0.00‐0.67)	1.00	0.33 (0.00‐0.80)
Patient involvement				
Introduction of clinicians involved in handover to patient/carer	0.84 (0.73‐0.94)	0.71 (0.55‐0.87)	1.00	0.83 (0.71‐0.93)
Invitation for patient/carer to participate in handover	0.82 (0.69‐0.92)	0.65 (0.44‐0.83)	0.94 (0.78‐1.00)	0.77 (0.59‐0.89)

a 95% CIs are not provided for labels with few positive examples in the test set.

b Accuracy: (true positives + true negatives)/all evaluated transcripts for that checklist item.

### Uncertainty and Unknown-Fact Span Extraction

Broad uncertainty-span extraction included all annotated uncertainty categories (hedging, vagueness, unknown facts, indefinite timing, source uncertainty, procedural uncertainty, and responsibility uncertainty) and had the lowest micro*–F*_1_-score of the evaluated tasks. The highest DSPy/GEPA point estimate used GPT-5.2 and achieved a precision of 0.32 (95% CI 0.26‐0.39), a recall of 0.56 (95% CI 0.44‐0.67), a micro*–F*_1_-score of 0.41 (95% CI 0.33‐0.48), and a mean IoU of 0.83 (95% CI 0.77‐0.90). The highest unoptimized-prompt baseline had a micro*–F*_1_-score of 0.35 (95% CI 0.28‐0.41), and the highest LangExtract uncertainty result had a micro*–F*_1_-score of 0.24 (95% CI 0.17‐0.31).

The narrower unknown-fact subtask had higher point estimates. DSPy/GEPA reached a micro*–F*_1_-score of 0.84 (95% CI 0.63‐1.00) with GPT-5.2, compared with the highest baseline micro*–F*_1_-score of 0.76 (95% CI 0.50‐1.00) and the LangExtract GPT-5.2 micro*–F*_1_-score of 0.56 (95% CI 0.29‐0.72). These results concern explicitly stated lack of knowledge, not facts absent from the transcript. Table 6 summarizes the uncertainty and unknown-fact span results used for these comparisons.

**Table 6.** Uncertainty and unknown-fact span extraction.

Task, model, and approach	Precision (95% CI)	Recall (95% CI)	*F*_1_-score (95% CI)	Mean IoU^a^ (95% CI)
Uncertainty				
GPT-5.2				
Baseline	0.24 (0.19‐0.29)	0.65 (0.53‐0.75)	0.35 (0.28‐0.41)	0.62 (0.53‐0.70)
DSPy^b^/GEPA^c^	0.32 (0.26‐0.39)	0.56 (0.44‐0.67)	0.41 (0.33‐0.48)	0.83 (0.77‐0.90)
LangExtract	0.35 (0.21‐0.55)	0.07 (0.03‐0.13)	0.12 (0.05‐0.20)	0.35 (0.28‐0.43)
MedGemma 27B				
Baseline	0.12 (0.07‐0.18)	0.17 (0.10‐0.23)	0.14 (0.08‐0.20)	0.56 (0.39‐0.70)
DSPy/GEPA	0.19 (0.12‐0.31)	0.20 (0.13‐0.28)	0.20 (0.13‐0.27)	0.72 (0.55‐0.88)
LangExtract	0.22 (0.15‐0.29)	0.26 (0.18‐0.36)	0.24 (0.17‐0.31)	0.69 (0.57‐0.80)
Unknown fact				
GPT-5.2				
Baseline	0.73 (0.44‐1.00)	0.80 (0.56‐1.00)	0.76 (0.50‐1.00)	0.86 (0.66‐0.99)
DSPy/GEPA	0.89 (0.71‐1.00)	0.80 (0.56‐1.00)	0.84 (0.63‐1.00)	0.92 (0.74‐0.99)
LangExtract	0.41 (0.17‐0.59)	0.90 (0.79‐1.00)	0.56 (0.29‐0.72)	0.68 (0.45‐0.84)
GPT-5-nano				
Baseline	0.08 (0.00‐0.17)	0.40 (0.00‐1.00)	0.13 (0.00‐0.27)	0.74 (0.00‐0.97)
DSPy/GEPA	0.89 (0.71‐1.00)	0.80 (0.56‐1.00)	0.84 (0.63‐1.00)	0.89 (0.79‐0.97)
MedGemma 27B				
Baseline	0.30 (0.09‐0.48)	0.80 (0.56‐1.00)	0.43 (0.16‐0.61)	0.90 (0.72‐0.98)
DSPy/GEPA	0.80 (0.67‐1.00)	0.80 (0.56‐1.00)	0.80 (0.63‐1.00)	0.90 (0.72‐0.98)

a IoU: intersection over union for matched span boundaries.

b DSPy: framework for declarative language model programming.

c GEPA: Genetic-Pareto.

## Discussion

### Principal Results

This study identified that prompt optimization with GEPA outperformed baseline evaluations across all of the comparisons, with higher aggregate performance for checklist prediction than for SBAR span extraction. This pattern is expected because using generative AI to perform clinical natural language processing tasks is sensitive to framing, label definitions, and example selection. GEPA was explicitly supplied with task-specific scores and error feedback that could align instructions more closely with the annotation scheme [13,23]. Optimization was particularly useful for improving targeted span extraction across the SBAR task.

The difference in accuracy that we identified between checklist prediction and SBAR span extraction provides an important insight to consider for designing AI applications to support clinical handover. Checklist prediction is a simpler structured-output task where each item is a bounded present/absent judgment. By contrast, SBAR extraction requires the model to locate clinically relevant text, assign a communicative category, and reproduce appropriate span boundaries. Structured handover tools aim to make minimum content predictable and reduce omissions, while still requiring adaptation to local clinical workflow [25-27]. As such, bounded present/absent outputs may be useful when the intended function is to prompt review of missing elements or support audit and quality monitoring. It is fortunate, then, that the task with the strongest performance in our study was the one most closely aligned with a recurring mechanism of handover-related harm, namely omitted or incomplete information [2,26,28]. Although checklist prediction achieved high accuracy, the consequences of both false positives and false negatives should be considered. A false negative would usually create review burden by prompting clarification of an item that was already communicated, but a false positive could create more serious false reassurance that a safety-critical element was conveyed when it was not. In addition, a checklist item not detected in the captured transcript is not equivalent to clinically necessary information having been omitted. A safer near-term design could therefore present AI outputs as source-linked prompts for clinician review, with clear distinctions between detected in transcript, not detected, and requires verification. Evidence has indicated that effective clinical decision support is most useful when integrated into a workflow as actionable and readily available support at the time and place of decision-making, while also recognizing the risk of automation bias when clinicians overrely on system outputs [29-31].

Registered nurse annotation agreement was comparatively high for checklist and SBAR annotations but very low for the broad uncertainty taxonomy. The latter finding indicates that vague, hedged, or context-dependent communication was difficult to operationalize consistently even with written guidance. However, the combination of low κ and relatively high matched IoU scores for broad uncertainty indicates that the principal difficulty was deciding whether uncertainty was present, rather than locating its boundaries once identified. As all model optimization and evaluation used the final consensus ratings between annotators, the results should therefore be interpreted as being conservative.

This study did not compare LLMs with non–LLM-based methods of structured information extraction. It therefore provides evidence about relative prompt optimization approaches and model configurations, not the superiority of LLMs over conventional information extraction. Nevertheless, evaluating LLM-based approaches is a logical next step in this handover-specific research program. In the original NICTA benchmark, a feature-engineered conditional random field achieved a macro*–F*_1_-score of 0.702 across 35 handover-form categories, but performance was markedly uneven. *F*_1_-score was 0.217 for the more abstract “other observations” category and 0.496 for future-care goals, tasks, and expected outcomes, and the error analysis identified clinically relevant missed and misclassified information [17]. These limitations were concentrated in categories requiring greater contextual differentiation. Instruction-tuned LLMs therefore warranted evaluation as a potentially more flexible approach to context-dependent handover information, particularly where prompt optimization can adapt extraction behavior using a modest labeled set.

The optimized SBAR extraction model achieved high span-boundary overlap among matched spans in our study. It should be considered, though, that SBAR is an idealized communication framework, while real clinical handovers often move nonlinearly, revisit information, distribute relevant details across the conversation, or place content between categories. For implementation, this suggests that extracted spans could be displayed with a link to their source transcript context and should support clinician review of what was said, rather than automatically transforming the handover into a definitive structured note. This preserves the benefits of structured communication while avoiding over-compression of clinically meaningful narrative context [25,32,33].

### Implementation Considerations

The appropriate balance between recall and precision may differ by use case when considering the implementation of the tasks we evaluated in this study into an AI tool to support clinical handover. For real-time clinician support, the intervention should be framed as shaping safe handover dialogue rather than only producing a structured output after the exchange has ended. Higher recall may be appropriate where the system displays nondetected checklist items under an “Items to check” heading and allows the receiving clinician to mark each item as addressed, not relevant, or requiring clarification. This presentation would avoid implying that the information was definitely omitted while supporting clarification before responsibility transfers. For retrospective audit or compliance monitoring, precision becomes more important because false positives could overestimate handover quality and obscure residual safety risks. The present checklist results, with high recall but nontrivial false positives in several categories, therefore support cautious separation of 2 implementation pathways: real-time clinician-facing gap prompts and separately validated audit/reporting workflows [28,29,34].

Gold standard handovers include active verification and shared situational awareness rather than passive transfer of uncertain information [33,35-37]. Evaluation results of the broad uncertainty-span performance indicate that even a GEPA-optimized prompt with a state-of-the-art proprietary LLM is not yet reliable for detecting the full range of ambiguous, hedged, or context-dependent communication. This should be interpreted against the clinical reality that uncertainty is also difficult for humans to recognize and act on consistently, particularly under time pressure or when concern is communicated indirectly. Many forms of clinically important uncertainty are implicit and embedded in shared team understanding: phrases such as “he seems off today” or “not quite themself” can convey concern through context, trajectory, and prior knowledge rather than through explicit wording that a model can reliably extract. By contrast, the comparatively more accurate unknown-fact extraction results suggest a potentially useful role for identifying explicit information gaps that can be converted into clarification prompts for the receiving clinician. As foundation models continue to improve, accuracy on this task may also improve, although reliable use in safety-critical handover settings will still require ongoing empirical validation.

Relatedly, it is highly likely that further gains could be realized after implementation independent of general improvements in underlying LLM capability. If clinicians review AI-generated handover outputs and corrections are retained as labeled examples, subsequent optimization cycles using the same GEPA prompt optimization pipeline we used in this study would plausibly improve performance over time. Prior clinical natural language processing active-learning studies have demonstrated that selectively labeled examples can improve data efficiency for both clinical text classification and clinical named-entity recognition [38,39]. In practice, any such learning cycle should be treated as a controlled quality-improvement process, with monitoring and re-evaluation before revised prompts or models are released into clinical use [40].

Finally, it should be noted that this study measured extraction and classification performance against annotated labels but did not evaluate whether AI outputs changed clinician questioning, closed-loop communication, task completion, escalation, near-miss detection, or patient outcomes. Technical metrics are necessary but insufficient for determining whether a clinical AI tool improves safety in practice [31,37,41]. Recent commentary and discursive work on nursing automation similarly argue that evaluation should move beyond time saved to examine how AI redistributes nursing work toward review and verification, whether tools meet end user–defined use thresholds, whether performance is equitable across linguistic and workforce groups, and how automation can be integrated without undermining professional values or relational care [42,43]. Even accurate prompts may create new risks if clinicians overtrust them, ignore nonhighlighted issues, experience alert fatigue, or redirect attention away from direct patient/carer engagement. Human factors testing should therefore assess reliance, trust calibration, interruption burden, and the usability of source-linked evidence before deployment [30,36,44].

### Limitations

The synthetic conversational handovers used in this study may not fully represent those performed in actual clinical practice where interruptions, time pressure, environmental noise, nonverbal cues, and local team dynamics can affect what is communicated and how it is interpreted. External validation on real handover audio/transcripts from intended deployment settings is therefore required [33,35,44]. External validity may be particularly limited for complex areas such as mental health, multimorbidity, and other contexts where clinical risk is often communicated through narrative nuance, formulation, behavioral change, staff concern, or tone of interaction rather than discrete data points. These contexts strengthen the need for AI outputs to remain clinician-reviewed, source-linked supports rather than autonomous clinical documentation.

It should be noted that several checklist items had low prevalence in the evaluation partition. Future evaluation should oversample or otherwise specifically test these items [26,31]. Furthermore, the reference standard retained only annotations agreed by both reviewers. This creates a conservative and reproducible benchmark but may exclude ambiguous or contested communication.

The evaluation corpus included synthetic transcripts generated in part with GPT-5. Although the uses were distinct, information extraction tasks were performed on synthesized language from a model from the same LLM provider, which is potentially susceptible to model familiarity. Synthetic transcripts may also be more predictable than authentic handover communication. In addition, the highest-performing models evaluated in this study were hosted proprietary systems that are updated and managed externally. Model updates, infrastructure variability, or configuration changes could alter performance over time in ways that are not fully transparent or controllable, with implications for reproducibility, governance, and consistency in clinical settings where reliability is critical. Locally hosted models may offer greater control over model versioning and deployment conditions but can require substantial compute and may be less responsive to rapid improvements in frontier model capability.

Our evaluation focused on downstream extraction from transcripts and did not separately quantify transcription accuracy, speaker attribution, or the effect of noisy audio. In a real-time handover system, errors introduced before the LLM stage could alter checklist detection, SBAR span extraction, and uncertainty identification. End-to-end evaluation should therefore include audio capture, transcription, diarization, and transcript-to-output performance under realistic clinical conditions [2,44]. We used a checklist that was developed at a local institution based on local priorities for communication during clinical nursing handover. Other wards, specialties, transfer types, or jurisdictions may prioritize different minimum datasets or use different terminology. Implementation would therefore require local mapping of labels to existing handover policy, audit tools, escalation pathways, and documentation workflows, followed by local validation rather than direct transfer of the reported performance estimates [26,34,44].

### Conclusions

In this evaluation of AI-assisted clinical handover tasks, prompt optimization consistently improved matched model performance. Accuracy was the highest for bounded checklist prediction and SBAR span extraction. Broad uncertainty detection remained insufficiently reliable, while explicit unknown-fact extraction suggests a narrower but important role for prompting clarification when missing knowledge is directly expressed. Before clinical deployment, these approaches require external validation on real handover audio and transcripts; end-to-end testing of transcription and diarization effects; local calibration of checklist definitions, human-factors evaluation of trust, reliance, workflow burden, and downstream safety outcomes; and structured assessment of organizational readiness for AI implementation in nursing care [45].

## Supplementary material

10.2196/106133 Checklist 1 TRIPOD-LLM checklist.

## Abbreviations

GEPA: Genetic-Pareto
IoU: intersection over union
LLM: large language model
NICTA: National Information and Communications Technology Australia
SBAR: Situation, Background, Assessment, Recommendation
TRIPOD-LLM: Transparent Reporting of a Multivariable Prediction Model for Individual Prognosis or Diagnosis–Large Language Models

## References

[1] (2026). Further information on communicating for safety. Australian Commission on Safety and Quality in Health Care.

[2] Ong MS, Coiera E (2011). A systematic review of failures in handoff communication during intrahospital transfers. Jt Comm J Qual Patient Saf.

[3] Manias E, Geddes F, Watson B, Jones D, Della P (2016). Perspectives of clinical handover processes: a multi-site survey across different health professionals. J Clin Nurs.

[4] The Joint Commission, and Joint Commission International (2007). Communication during patient hand-overs. https://cdn.who.int/media/docs/default-source/patient-safety/patient-safety-solutions/ps-solution3-communication-during-patient-handovers.pdf.

[5] Hada A, Jones LV, Jack LC, Coyer F (2021). Translating evidence-based nursing clinical handover practice in an acute care setting: a quasi-experimental study. Nurs Health Sci.

[6] Yung AHW, Pak CS, Watson B (2023). A scoping review of clinical handover mnemonic devices. Int J Qual Health Care.

[7] McCarthy S, Motala A, Lawson E, Shekelle PG (2025). Use of structured handoff protocols for within-hospital unit transitions: a systematic review from Making Healthcare Safer IV. BMJ Qual Saf.

[8] Moyo P, Anderson J, Francis K, Biles J (2024). Exploring the experiences and perceptions of the utilisation of structured clinical handover frameworks by nurses working in acute care settings: a scoping review. J Clin Nurs.

[9] Chien LJ, Slade D, Goncharov L (2024). Implementing a ward-level intervention to improve nursing handover communication with a focus on bedside handover-a qualitative study. J Clin Nurs.

[10] Tobiano G, Bucknall T, Sladdin I, Whitty JA, Chaboyer W (2018). Patient participation in nursing bedside handover: a systematic mixed-methods review. Int J Nurs Stud.

[11] Anshasi H, Almayasi ZA (2024). Perceptions of patients and nurses about bedside nursing handover: a qualitative systematic review and meta-synthesis. Nurs Res Pract.

[12] Agrawal M, Hegselmann S, Lang H, Kim Y, Sontag D (2022). Large language models are few-shot clinical information extractors. Proc 2022 Conf Empir Methods Nat Lang Process.

[13] Sivarajkumar S, Kelley M, Samolyk-Mazzanti A, Visweswaran S, Wang Y (2024). An empirical evaluation of prompting strategies for large language models in zero-shot clinical natural language processing: algorithm development and validation study. JMIR Med Inform.

[14] Agha-Mir-Salim L, Alberto IR, Alberto NR (2025). Technological solutions to improve inpatient handover in the era of artificial intelligence: scoping review. J Med Internet Res.

[15] Chen RJ, Wu MS, Tsai LW, Chang SS, Shen Hsiao ST, Lo YS (2026). Integrating a large language model to streamline nursing handover documentation across multiple hospitals in Taiwan: development and implementation study. J Med Internet Res.

[16] Gallifant J, Afshar M, Ameen S (2025). The TRIPOD-LLM reporting guideline for studies using large language models. Nat Med.

[17] Suominen H, Zhou L, Hanlen L, Ferraro G (2015). Benchmarking clinical speech recognition and information extraction: new data, methods, and evaluations. JMIR Med Inform.

[18] Khattab O, Singhvi A, Maheshwari P DSPy: compiling declarative language model calls into self-improving pipelines. https://openreview.net/pdf?id=sY5N0zY5Od.

[19] Australian Research Council (ARC); Universities Australia (2025). National statement on ethical conduct in human research 2025. https://www.nhmrc.gov.au/sites/default/files/documents/attachments/publications/National-Statement-on-Ethical-Conduct-Human-Research-2025.pdf.

[20] Prodigy.

[21] Singh A, Fry A, Perelman A (2025). OpenAI GPT-5 system card. arXiv.

[22] Sellergren A, Kazemzadeh S, Jaroensri T (2025). MedGemma technical report. arXiv.

[23] Agrawal LA, Tan S, Soylu D GEPA: reflective prompt evolution can outperform reinforcement learning. https://openreview.net/pdf?id=RQm2KQTM5r.

[24] Goel A (2026). LangExtract v1.7.0. Zenodo.

[25] Haig KM, Sutton S, Whittington J (2006). SBAR: a shared mental model for improving communication between clinicians. Jt Comm J Qual Patient Saf.

[26] Riesenberg LA, Leitzsch J, Cunningham JM (2010). Nursing handoffs: a systematic review of the literature. Am J Nurs.

[27] Bukoh MX, Siah CJR (2020). A systematic review on the structured handover interventions between nurses in improving patient safety outcomes. J Nurs Manag.

[28] Manser T, Foster S (2011). Effective handover communication: an overview of research and improvement efforts. Best Pract Res Clin Anaesthesiol.

[29] Kawamoto K, Houlihan CA, Balas EA, Lobach DF (2005). Improving clinical practice using clinical decision support systems: a systematic review of trials to identify features critical to success. BMJ.

[30] Goddard K, Roudsari A, Wyatt JC (2012). Automation bias: a systematic review of frequency, effect mediators, and mitigators. J Am Med Inform Assoc.

[31] Challen R, Denny J, Pitt M, Gompels L, Edwards T, Tsaneva-Atanasova K (2019). Artificial intelligence, bias and clinical safety. BMJ Qual Saf.

[32] Rosenbloom ST, Denny JC, Xu H, Lorenzi N, Stead WW, Johnson KB (2011). Data from clinical notes: a perspective on the tension between structure and flexible documentation. J Am Med Inform Assoc.

[33] Cohen MD, Hilligoss PB (2010). The published literature on handoffs in hospitals: deficiencies identified in an extensive review. Qual Saf Health Care.

[34] Redley B, Waugh R (2018). Mixed methods evaluation of a quality improvement and audit tool for nurse-to-nurse bedside clinical handover in ward settings. Appl Nurs Res.

[35] Patterson ES, Roth EM, Woods DD, Chow R, Gomes JO (2004). Handoff strategies in settings with high consequences for failure: lessons for health care operations. Int J Qual Health Care.

[36] Leonard M, Graham S, Bonacum D (2004). The human factor: the critical importance of effective teamwork and communication in providing safe care. Qual Saf Health Care.

[37] Starmer AJ, Spector ND, Srivastava R (2014). Changes in medical errors after implementation of a handoff program. N Engl J Med.

[38] Figueroa RL, Zeng-Treitler Q, Ngo LH, Goryachev S, Wiechmann EP (2012). Active learning for clinical text classification: is it better than random sampling?. J Am Med Inform Assoc.

[39] Chen Y, Lasko TA, Mei Q, Denny JC, Xu H (2015). A study of active learning methods for named entity recognition in clinical text. J Biomed Inform.

[40] Feng J, Phillips RV, Malenica I (2022). Clinical artificial intelligence quality improvement: towards continual monitoring and updating of AI algorithms in healthcare. NPJ Digit Med.

[41] Kelly CJ, Karthikesalingam A, Suleyman M, Corrado G, King D (2019). Key challenges for delivering clinical impact with artificial intelligence. BMC Med.

[42] Ronquillo CE (2026). Beyond time saved: implementation, equity, and the utility threshold for nursing AI scribes. J Med Internet Res.

[43] Pepito JA, Acaso NJ, Merioles R, Ismael J (2025). Opportunities, challenges, and future directions for the integration of automation in nursing practice: discursive study. JMIR Nurs.

[44] Sittig DF, Singh H (2010). A new sociotechnical model for studying health information technology in complex adaptive healthcare systems. Qual Saf Health Care.

[45] Seibert K, Domhoff D, Altona J (2026). Readiness assessment for AI in nursing care projects: multimethods study. JMIR Nurs.

[46] Large language models for structured information extraction in artificial intelligence-assisted clinical handover: evaluation study. Figshare.

