# Supplementary material for: AI-Based Structured Information Extraction From Synthetic Nursing Handover Transcripts: Comparative Evaluation of Large Language Models
Source: JMIR Nurs. 2026 Oct 5;9:e106133. doi: 10.2196/106133 (PMC13638071; doi:10.2196/106133)
Supplement: Supplementary file 1 [file nursing-v9-e106133-s001.pdf]

TRIPOD-LLM Checklist

Completed for: Large Language Models for Structured Information Extraction in Artificial Intelligence-Assisted Clinical Handover: Evaluation Study

Section	Item	Checklist item	Research design	LLM task	Page(s)	Reporting location / notes
Title and abstract	1	Identify the study as developing, fine-tuning, and/or evaluating the performance of a large language model, specifying the task, target population, and outcome to be predicted.	All	All	p 1	Title identifies LLM evaluation, structured information extraction, clinical handover, and evaluation study design.
Title and abstract	2	Abstract: see TRIPOD-LLM for Abstracts.	All	All	pp 2-4	Structured abstract reports background, objective, methods, results, and conclusions, including tasks, models, dataset, and main performance estimates.
Background	3a	Explain the healthcare context or use case and rationale for developing and/or evaluating the LLM, including references to existing approaches or models.	All	All	pp 4-5	Introduction explains clinical handover, SBAR, communication risk, and the rationale for LLM-assisted handover support.
Background	3b	Describe the target population and intended use of the LLM in the care pathway, including intended users in current gold-standard practices.	E/H	All	pp 5, 17-18	Methods describe simulated nursing handover transcripts and intended handover-support tasks; Discussion describes clinician oversight and implementation considerations.
Background	4	Specify the objectives, including whether the study concerns initial development, fine-tuning, or validation/evaluation of an LLM.	All	All	pp 4-5	Objective is evaluation of LLM performance and prompt optimisation for structured information extraction in clinical handover.
Methods: Data	5a	Describe data sources separately for training, tuning, and evaluation, and give rationale for their use.	All	All	pp 6-7, 9	Data sources section describes NICTA synthetic data, institutional educational videos, additional synthetic examples, and deterministic optimisation/evaluation split.
Methods: Data	5b	Describe relevant data points and give quantitative or qualitative descriptions of their distributions and descriptors.	All	All	pp 6-7, 31-36	Dataset size and clinical scenario sources are reported; checklist and span labels are summarised in Tables 1-2 and performance tables.
Methods: Data	5c	Report dates of the oldest and newest text used in development and evaluation datasets.	All	All	pp 6-7	Data sources are described
Methods: Data	5d	Describe data preprocessing and quality checking, and whether these were similar across corpora, institutions, or groups.	All	All	pp 6-8, 11-12	Methods describe Whisper transcription, synthesis workflow, annotation, consensus reference standard, validation of few-shot examples, and output normalisation.
Methods: Data	5e	Describe handling of missing or imbalanced data and reasons for omitting data.	All	All	pp 8, 12-13	Consensus-only reference labels and bootstrap performance estimation are described; no clinical records or participant observations were omitted.
Methods: Analytical methods	6a	Specify the LLM name, version, and last date of training.	All	All	p 9	Models are named as GPT-5.2, GPT-5-nano, and MedGemma 27B; citations provide model documentation. Training cut-off dates are not fully reported in the manuscript.
Methods: Analytical methods	6b	Describe relevant LLM development details, such as architecture, training, fine-tuning, alignment strategy, and goals.	M/D	All	p 9	The study evaluates existing models rather than developing a de novo LLM. Public/open-weight model documentation is cited where available.
Methods: Analytical methods	6c	Describe text generated using LLMs, prompt engineering, and inference settings.	M/D/E	All	pp 9-12	Methods describe DSPy signatures, baseline prompting, GEPA prompt optimisation, LangExtract prompts, few-shot examples, and normalised structured outputs.
Methods: Analytical methods	6d	Describe the initial and post-processed output.	All	All	pp 9-12	Outputs were constrained structured checklist labels or label-and-verbatim-quote pairs, then normalised and mapped back to transcripts for scoring.
Methods: Analytical methods	6e	For classification or outcome forecasting, describe probabilities, thresholds, and rationale.	All	C/OF	pp 9, 12-13, 34-35	Checklist prediction is multilabel classification scored against consensus labels; no probability-threshold output is reported.
Methods: LLM output	7a	Describe metrics used to capture the quality of generative outputs, such as consistency, relevance, and accuracy against gold standards.	All	QA/IR/DG/SS/MT	pp 12-13, 33-36	Accuracy, precision, recall, F1, same-label quote matching, and mean IoU are reported against consensus reference labels.
Methods: LLM output	7b	Explain relevance of outcome metrics to the downstream task at deployment time and correlation with human evaluation if applicable.	E/H	All	pp 12-13, 17-18	Metrics reflect content verification and span detection needed for handover support; Discussion interprets findings in relation to clinical handover use.

Section	Item	Checklist item	Research design	LLM task	Page(s)	Reporting location / notes
Methods: LLM output	7c	Define the outcome, how LLM predictions were calculated, date of inference for closed-source LLMs, and evaluation metrics.	E/H	All	pp 9-13, 33-36	Outcomes and metrics are defined; closed-source inference dates are not reported.
Methods: LLM output	7d	If subjective interpretation was used, report assessor qualifications, instructions, demographics, and inter-assessor agreement.	All	All	p 8, 31-32	Two clinically active registered nurses annotated all transcripts using written guidance; consensus labels were used. Inter-rater reliability was not calculated.
Methods: LLM output	7e	Describe how performance was compared with other LLMs, humans, benchmarks, or standards.	All	All	pp 9-15, 30, 33-36	Baseline, DSPy/GEPA, LangExtract, GPT-5.2, GPT-5-nano, and MedGemma configurations are compared using prespecified metrics.
Methods: Annotation	8a	If annotation was performed, describe how text was labeled and provide annotation guidelines or examples.	All	All	p 8, 31-32	Annotation process and interfaces are described; Tables 1-2 provide label categories and guidance/examples.
Methods: Annotation	8b	Report number of annotators, proportion annotated by more than one annotator, and inter-annotator agreement.	All	All	p 8	Two registered nurses independently annotated all transcripts; inter-rater reliability was not calculated because consensus labels were the reference standard.
Methods: Annotation	8c	Report background and experience of annotators or model characteristics used for annotation.	All	All	p 8	Annotators are described as experienced clinically active registered nurses.
Methods: Prompting	9a	Describe prompt design, curation, and selection.	All	All	pp 9-11	DSPy signatures, GEPA optimisation, task-specific feedback, and LangExtract prompt descriptions/few-shot examples are described.
Methods: Prompting	9b	Describe data used to develop prompts.	All	All	pp 7, 9-11	Optimisation partition and annotated few-shot examples from reference data were used for prompt development and evaluation workflows.
Methods: Summarization	10	For summarization tasks, describe preprocessing before summarization.	All	SS	N/A	The study did not evaluate summarization or simplification tasks.
Methods: Instruction tuning/alignment	11	If instruction tuning or alignment strategies were used, describe instructions, data, interface, and population characteristics.	M/D	All	N/A	The study used prompt optimisation and few-shot prompting, not instruction tuning or model alignment.
Methods: Compute	12	Report compute or proxies for compute required for the methods.	M/D/E	All	pp 9-10	Cloud-hosted OpenAI models were run using the OpenAI API; MedGemma 27B was run on institutional high-performance computing infrastructure. GEPA optimisation scoring-call count is also reported.
Methods: Ethical approval	13	Report ethics committee or institutional review board approval and consent/waiver details.	All	All	p 8	Ethics approval was not sought because only synthetic and simulated handover transcripts were used.
Methods: Open science	14a	Report funding source and role of the funder.	All	All	p 22	Funding source is stated as The Prince Charles Hospital Foundation Collaboration Grant; manuscript includes a placeholder to confirm funder role.
Methods: Open science	14b	Report conflicts of interest and financial disclosures.	All	All	p 22	Authors declare no conflicts of interest.
Methods: Open science	14c	Report protocol accessibility or state that no protocol is available.	H	All		Protocol from grant application is available.
Methods: Open science	14d	Report registration information or state that the study was not registered.	H	All		Not registered
Methods: Open science	14e	Report study data availability.	All	All	p 22	Data availability statement is included, with a repository URL placeholder.
Methods: Open science	14f	Report code availability.	All	All	Not reported	Code availability on request.
Methods: Public involvement	15	Describe patient and public involvement or state no involvement.	H	All	p 5	Methods state that tasks were identified through a co-design process with clinicians and consumers that will be reported separately.
Results: Participants/data	16a	For patient/EHR data, report the flow of text, EHR, or patient data through the study, including counts.	E/H	All	N/A; pp 6-7	No patient/EHR data were used. Synthetic and simulated transcript sources and final sample size are reported.
Results: Participants/data	16b	Report characteristics overall and by source, setting, split, key dates, and sample size.	E/H	All	pp 6-7, 31-32	Data source, setting, final sample size, and label categories are reported; exact dates are not fully reported.
Results: Participants/data	16c	For clinical outcomes, compare distribution of important clinical variables between development and evaluation data.	E/H	All	N/A	The study did not predict clinical outcomes or use real patient clinical variables.
Results: Participants/data	16d	For patient/EHR data, report number of participants and outcome events in each analysis.	E/H	All	N/A	No patient/EHR participants were included.

Section	Item	Checklist item	Research design	LLM task	Page(s)	Reporting location / notes
Results: Performance	17	Report LLM performance according to prespecified metrics and human evaluation, where applicable.	All	All	pp 13-15, 30, 33-36	Results text, Figure 1, and Tables 3-5 report performance across models, tasks, and prompt strategies.
Results: LLM updating	18	If applicable, report updated LLM and performance.	All	All	N/A	No model updating after evaluation was performed.
Discussion: Interpretation	19a	Provide overall interpretation of main results, including fairness where relevant, in context of objectives and prior studies.	All	All	pp 15-17	Discussion interprets model performance for handover support and relates findings to prior LLM and nursing documentation work.
Discussion: Limitations	19b	Discuss limitations and effects on bias, statistical uncertainty, and generalizability.	All	All	pp 19-20	Limitations discuss synthetic data, sample size, prompt/model scope, and generalizability.
Discussion: Usability in context	19c	Discuss known challenges using data for the task/domain, including representation, missingness, harmonization, and bias.	E/H	All	pp 19-20	Limitations discuss synthetic transcripts and need for validation in real handover environments.
Discussion: Usability in context	19d	Describe intended implementation use, intended input, end user, autonomy, and human oversight.	E/H	All	pp 17-18	Implementation considerations frame the work as clinician-facing handover support requiring empirical validation and human oversight.
Discussion: Usability in context	19e	Discuss handling of poor-quality or unavailable input data when implementing the LLM.	E/H	All	pp 19-20	Limitations note need for testing in real audio/transcription and clinical contexts; detailed failover procedures are not yet specified.
Discussion: Usability in context	19f	Describe user interaction in input handling or use, and expertise required.	E/H	All	pp 17-18	Discussion positions outputs as support for receiving clinicians and targeted clarification rather than autonomous decision-making.
Discussion: Usability in context	19g	Describe future research needed for applicability and generalizability.	All	All	pp 19-21	Limitations and conclusions identify need for larger evaluations, real-world data, and clinical validation.

Abbreviations: C=classification; D=de novo LLM development; DG=document generation; E=LLM evaluation; EHR=electronic health record; H=LLM evaluation in healthcare settings; IR=information retrieval; LLM=large language model; M=LLM methods; MT=machine translation; OF=outcome forecasting; QA=long-form question-answering; SS=summarization and simplification.

Note: Items marked N/A do not apply to this evaluation-only study using synthetic and simulated handover transcripts. Items marked not reported or partial indicate places where the current manuscript does not fully address a TRIPOD-LLM item.
